# ANAMT technical guideline: occupational telediagnosis

**DOI:** 10.47626/1679-4435-2023-1226

**Published:** 2023-11-24

**Authors:** Francisco Cortes Fernandes, Rosylane Nascimento das Mercês Rocha, Mírian Pérpetua Palha Dias Parente, Ricardo de Almeida Sebba, Leonardo Pereira Cabral, Wanderley Marques Bernardo

**Affiliations:** 1 Núcleo Diretrizes, Associação Nacional de Medicina do Trabalho, São Paulo, SP, Brazil; 2 Programa Diretrizes, Associação Médica Brasileira, São Paulo, SP, Brazil

**Keywords:** telemedicine, medical examination, occupational health, practice guideline, telemedicina, exames médicos, saúde ocupacional, guia de prática clínica

## Abstract

The relationships between work and health/illness are the main task of the occupational physician, with the occupational medical examination being used to address these relationships, together with a workplace study and epidemiological analyses. This study had as a guiding clinical question: Is telemedicine occupational examination (telediagnosis) accurate compared with in-person occupational examination? The studies were selected by four independent reviewers, meeting the eligibility criteria. The searches resulted in 12,654, 29, 3, and 0 articles retrieved from MEDLINE, EMBASE, and Google Scholar databases and hand search, respectively. Of this total, 284 studies were selected by title and abstract screening, none of which met the previously established eligibility criteria for study inclusion (references excluded). There is currently no evidence comparing regular or standard (in-person) occupational examination vs telemedicine occupational examination. Therefore, there is no supporting evidence to recommend the use of occupational telediagnosis (occupational examination).

## Abstract (pt)

As relações entre trabalho e saúde/doença são a principal tarefa do médico do trabalho, sendo o exame ocupacional utilizado para abordar essas relações, juntamente com o estudo do local de trabalho e análises epidemiológicas. Este estudo teve como questão clínica norteadora: o exame ocupacional por telemedicina (telediagnóstico) é acurado quando comparado ao exame ocupacional presencial? Os trabalhos foram selecionados por quatro revisores independentes, atendendo aos critérios de elegibilidade. Foram recuperados, nas bases consultadas MEDLINE, EMBASE, Google Scholar e manual, respectivamente, 12.654, 29, 3 e 0 artigos. Desse montante, foi selecionado pelo título e resumo um total de 284 estudos (referências excluídas), dos quais não foi possível selecionar nenhum que atendesse aos critérios de elegibilidade previamente estabelecidos. No momento, não há evidência comparando o exame ocupacional regular ou padrão (presencial) e o mesmo exame por meio de telemedicina. Portanto, não há como recomendar o uso de telediagnóstico ocupacional (exame ocupacional).

## INTRODUCTION

The relationships between work and health/illness are the main task of the occupational physician, with the occupational medical examination being the instrument used to address these relationships, together with a workplace study and epidemiological analyses.

Medical semiology is a branch of medicine that deals with the identification of the various manifestations of diseases and can be classified into two components: semiotechnique (technique for searching signs) and propaedeutics (which seeks to gather and interpret signs and symptoms to reach a diagnosis). Therefore, propaedeutics has the following structure: clinical and occupational history taking, physical examination, and diagnostic considerations.

The occupational medical examination is often seen simply as issuing a medical certificate, but we should understand and perform it seriously and extremely committed to its goals, given the potential repercussions on various judicial and ethical aspects.

An important characteristic of the occupational physician’s care is that occupational visits are usually not motivated by workers’ active search. Occupational visits are provided for by law, and a detailed history and physical examination can open up space for reporting possible diseases.

Finally, it is important to note that the occupational examination should aim not only to assess the worker’s health but also to verify the effectiveness of protection against the risks existing in the workplace and the impact caused by occupational exposures. Furthermore, it should analyze the worker’s health from a global point of view, unlike a clinical examination that aims to evaluate, diagnose, and treat a disease.

Thus, clinical examination is the main instrument in the practice of occupational medicine. A well-performed examination can produce data that can be transformed into information, which generates knowledge on the population of workers that can inform the occupational physician’s practice.

In summary, we can say that the occupational examination has the following main objectives:

- To allocate workers to job positions suited to their physical and mental health conditions;
- To assess workers’ health status;
- To solve medical problems detected in workers;
- To assess whether exposure to occupational risk factors is impacting workers’ health;
- To perform worker reallocation;
- To conduct epidemiological surveys;
- To promote workers’ health;
- To assess fitness for work;
- To comply with legal requirements that make this examination mandatory.

To achieve the objectives of occupational medicine when performing occupational examinations, we need to keep in mind the risks to which workers are exposed during work. This knowledge will allow the physician to truly identify their working conditions and become aware of the supervisors who lead these activities.

A careful occupational history taking is the most effective way to diagnose diseases, and it is important to ask workers about all tasks they perform and the duration of each task. It is not just about listing the tasks, it is also about including the duration and details of the activities, the use of personal protective equipment, in addition to hygiene and occupational safety practices. It is important to include the name of the job position in each task.

Medicine, understood as a science, requires the use of refined techniques to achieve its purpose. When taking a medical history, the technique is represented by the order and depth of the questions. A careful attitude on the part of physicians when performing the physical examination can increase their perception.

Due to legal and ethical aspects, it is recommended to perform a comprehensive physical examination to identify changes resulting from occupational exposure, which may go unnoticed in a focused examination.

It should also be remembered that the principles of physical examination are inspection, palpation, percussion, and auscultation.

## METHODS

### CLINICAL QUESTION

Is telemedicine occupational examination (telediagnosis) accurate compared with in-person occupational examination?

### ELIGIBILITY CRITERIA (STUDIES TO BE SELECTED)

- Patient: worker undergoing occupational examination
- Intervention: telemedicine physical examination
- Comparison: in-person physical examination
- Outcome: diagnosis or suspected diagnosis
- Study design: cross-sectional study
- Publication date: no restrictions
- Language: no restrictions
- Full text or abstract with data

### DATABASES SEARCHED AND SEARCH STRATEGY

The MEDLINE, EMBASE, and Google Scholar databases were searched for evidence.

#### Additional hand search

The following strategies were used:

- #1: (Employee OR Employment OR Labor OR Occupational OR Work OR Worker OR Working OR Workplace OR Workload)
- #2: (Physical Examination OR Physical Examinations OR Physical Exam OR Physical Exams)
- #3: (Telemedicine OR Tele-Referral OR Tele Referral OR Tele-Referrals OR Virtual Medicine OR Mobile Health OR mHealth OR Telehealth OR eHealth)
- #4: = #2 AND #3
- #5: = #1 AND #3 AND (sensitiv*[Title/Abstract] OR sensitivity and specificity[MeSH Terms] OR diagnose[Title/Abstract] OR diagnosed[Title/Abstract] OR diagnoses[Title/Abstract] OR diagnosing[Title/Abstract] OR diagnosis[Title/Abstract] OR diagnostic[Title/Abstract] OR diagnosis[MeSH:noexp] OR (diagnostic equipment[MeSH:noexp] OR diagnostic errors[MeSH:noexp] OR diagnostic imaging[MeSH:noexp] OR diagnostic services[MeSH:noexp]) OR diagnosis, differential[MeSH:noexp] OR diagnosis[Subheading:noexp]))
- FINAL: = #4 OR #5

### STUDY SELECTION

The studies were initially selected by screening titles and abstracts, then confirmed by full texts, by four independent reviewers, meeting the eligibility criteria.

### DATA EXTRACTION

Once the study is selected, the characteristics of the worker, the test (telemedicine physical examination), and the reference standard (in-person physical examination) will be extracted. The test accuracy measures will be the prevalence of identified changes, sensitivity, and specificity. If there are details, such as true positives and negatives and false positives and negatives, these will also be part of the extracted data.

### QUALITY OF EVIDENCE

The risk of bias in studies will be estimated using the QUADAS-2 tool,^1^ being graded as low, high, or very high. The quality of evidence will be directly extrapolated from this risk, being expressed as very low, low, or high.

### ANALYSIS AND EXPRESSION OF RESULTS

If the results of one or more studies are common, the data will be aggregated in a single analysis (meta-analysis) (Meta-DiSc software^2^) and expressed as sensitivity, specificity, and positive and negative predictive values.

### SUMMARY OF EVIDENCE

The available evidence will support the synthesis that will seek to answer the initial question of this review. The answer may be qualitative (in the absence of a meta-analysis) or quantitative (with meta-analyzed data).

## RESULTS

The searches resulted in 12,654, 29, 3, and 0 articles retrieved from MEDLINE, EMBASE, and Google Scholar databases and hand search, respectively. Of this total, 284 studies were selected by title and abstract screening, none of which met the previously established eligibility criteria for study inclusion (references excluded - Annex 1). The reasons for exclusion are explained in the tables in Annexes 2 to 5.

### SUMMARY OF EVIDENCE

There is currently no evidence comparing regular or standard (in-person) occupational examination vs telemedicine occupational examination. There is only scant evidence testing the use of telemedicine in isolated areas or topographies of the human body, such as skin, eyes, ears, mind, nervous and musculoskeletal systems. Therefore, there is no supporting evidence to recommend the use of occupational telediagnosis (occupational examination).

## References

[1] University of Bristol QUADAS-2. A quality assessment tool for diagnostic accuracy studies.

[2] Software Informer (2014). Meta-DiSc 1.4: a tool for performing meta-analysis of diagnostic and
screening tests.

